# Microstructural Analysis of Whey/Soy Protein Isolate Mixed Gels Using Confocal Raman Microscopy

**DOI:** 10.3390/foods10092179

**Published:** 2021-09-14

**Authors:** Laura G. Gómez-Mascaraque, Samantha C. Pinho

**Affiliations:** 1 Teagasc Food Research Centre, Food Chemistry & Technology Department, Moorepark, Fermoy, P61 C996 Cork, Ireland; 2 Department of Food Engineering, School of Animal Science and Food Engineering, University of Sao Paulo, Pirassununga 13635-900, Brazil; samantha@usp.br

**Keywords:** hyperspectral imaging, microscopy, microstructure, hydrogel, dairy, plant protein

## Abstract

This work explores the potential of confocal Raman microscopy to investigate the microstructure of mixed protein gel systems. Heat-set protein gels were prepared using whey protein isolate (WPI), soy protein isolate (SPI), and mixtures thereof, with a total of five different whey-to-soy protein ratios (100, 75, 50, 25, and 0%). These were analysed using confocal Raman microscopy, and different data analysis approaches were used to maximize the amount of structural and compositional information extracted from the spectral datasets generated, including both univariate and multivariate analysis methods. Small spectral differences were found between pure WPI and SPI gels, mainly attributed to conformational differences (amide bands), but SPI exhibited considerably greater auto-fluorescence than WPI. The univariate analysis method allowed for a rapid microstructural analysis, successfully mapping the distribution of protein and water in the gels. The greater fluorescence of the capsule-like structures found in the mixed gels, compared to other regions rich in proteins, suggested that these may be enriched in soy proteins. Further analysis, using a multivariate approach, allowed us to distinguish proteins with different levels of hydration within the gels and to detect non-proteinaceous compounds. Raman microscopy proved to be particularly useful to detect the presence of residual lipids in protein gels.

## 1. Introduction

Proteins play essential roles in food, not only as macronutrients, but also as structural ingredients, which aid in the formation of food gels and contribute to the desired texture and mouth-feel attributes of the final food products [1]. Among animal proteins, dairy proteins are highly nutritious and exhibit good techno-functional properties [2,3,4], including excellent gelling properties [5,6,7]. However, with the continuous growth in the world population, the demand for protein for human consumption is increasing [8], and a number of alternative sources are being explored to meet this demand [9,10,11,12]. Among them, plant proteins have received particular attention, mainly on the grounds of enhanced environmental sustainability [13,14,15]. The acceptance of plant proteins by consumers is also greater than for other alternative protein sources, such as insects or cultured meat [16].

The amino acid profile of plant proteins is generally less favourable than that of animal proteins [17], and the blend of several protein sources is usually required to ensure sufficient amounts of essential amino acids [18]. Moreover, mixing proteins from different sources may not only result in a more balanced amino acid uptake [19,20], but also in techno-functional synergies [21]. In this context, plant and animal protein blends are receiving increased interest in terms of the design of high protein food products, with soy and whey protein isolate mixtures being one of the most common combinations [22,23]. In fact, soy proteins have long been used to partially replace dairy proteins in several food products, including beverages [24], yogurts, and coffee creamers [25]. The microstructure of foodstuffs has a significant impact on their texture [26], overall sensory perception [27], and digestibility [28]. Bearing in mind that reformulation generally results in structural changes in food products, studying the microstructure of mixed protein systems is essential to gain a better understanding of the implications of partially replacing dairy proteins with plant proteins in food formulations.

Confocal Raman microscopy combines the principles of Raman spectroscopy with those of confocal microscopy and, thus, allows for the obtainment of both chemical and microstructural information of samples [29]. Compared to the more widely used confocal laser scanning microscopy (CLSM) technique that, for most applications in food, requires staining the samples with fluorescent dyes [30], Raman microscopy can identify the native components of specimens, without the need for labelling, through the analysis of the inelastic scattering of light [31,32]. Therefore, the microstructural analysis can be performed in a less invasive and less destructive manner, also avoiding potential artefacts derived from the addition of dyes, their solvents, or the staining protocols themselves [33]. Additionally, while the distribution of only a limited number of compounds of interest can be mapped simultaneously through CLSM in complex matrices (those that can be specifically stained with fluorophores whose emission spectra do not overlap), each pixel of a Raman micrograph contains chemical information from all the components present in that specific spot of the sample [34]. Due to its immense potential, new applications of Raman microscopy to study the microstructure of food are rapidly emerging [35,36,37].

The aim of this work was to explore the use of confocal Raman microscopy to investigate the microstructure of mixed protein gels and maximize the amount of structural and compositional information extracted from the spectral datasets generated by exploring different data analysis approaches, including both univariate and multivariate analysis methods. For this purpose, whey/soy mixed protein gels were used as a case study due to the industrial relevance of blends made from these proteins. Heat-set protein gels with different whey-to-soy protein ratios (100, 75, 50, 25, and 0%) were prepared and analysed through confocal Raman microscopy. Confocal laser scanning microscopy (CLSM) was also used as a reference method. The advantages and limitations of confocal Raman microscopy for the characterization of mixed protein gels are discussed, and the different approaches proposed for data analysis are compared.

## 2. Materials and Methods

### 2.1. Materials

Soy protein isolate (SPI), under the commercial name of Protimarti M-90 and with a protein content of 88%, was obtained from Marsul (Montenegro, RS, Brazil). Whey protein isolate (WPI), under the commercial name of Isolac and with a protein content of 91%, was purchased from Carbery (Cork, Ireland). Sodium chloride (NaCl) was acquired from Sigma Aldrich (Wicklow, Ireland).

### 2.2. Preparation of Heat-Set Protein Gels

WPI and SPI stock solutions (15 g/100 g) were prepared using methods adapted from Comfort and Howell [38]. The protein dispersions were prepared separately, hydrating the powder with deionized water and adding NaCl (final concentration 0.1 M). The pH was adjusted to 7.0 using a 1 M NaOH solution. The SPI dispersion was manually stirred, whereas the WPI dispersion was stirred magnetically at 800 rpm for 1 h. Both dispersions were kept under refrigeration overnight after preparation.

From the stock solutions, 5 mixed gels (15 g total protein/100 g gel) were prepared mixing various WPI:SPI mass ratios, i.e., 100/0 (WPI100), 75/25 (SPI25), 50/50 (SPI50), 25/75 (SPI75), and 0/100 (SPI100), stirred magnetically for 2 h at 600 rpm, and stored overnight under refrigeration for complete hydration. Afterwards, the protein dispersions were heated to 95 °C for 30 min and stored overnight under refrigeration before characterization.

### 2.3. Confocal Raman Microscopy (CRM)

#### 2.3.1. Specimen Preparation

Internal sections of the protein gels were cut with a blade, placed on glass microscopy slides, and covered with glass coverslips to avoid water evaporation. The space between the slide and the coverslip was sealed with BluTack adhesive to prevent drying of the hydrogels during analysis.

#### 2.3.2. Equipment and Software

The samples were analysed using an Alpha300 R confocal Raman microscope (WITec, Ulm, Germany) equipped with a 532 nm laser and an ultra-fast Raman imaging CCD camera. The Raman shift was calibrated using silicon. Project Five v5.0 and True Match software (WITec, Ulm, Germany) were used for image processing and analysis.

#### 2.3.3. Acquisition of Single Spectra

A 50× microscope objective (0.55 numerical aperture) was used to collect the spectra of the reference protein gels, i.e., the pure WPI and SPI gels. The spectrum of a glass coverslip was also collected. Each spectrum was collected with a laser power of 40 mW, an integration time of 1 s, and 20 accumulations, based on a preliminary optimization of these conditions. At least 5 spectra, collected at different random locations, were collected for each type of hydrogel and averaged. The raw spectra were processed using the cosmic ray removal correction function (filter size 3, dynamic factor 8) and a shape function for the background subtraction (shape size 250, noise factor 1).

#### 2.3.4. Confocal Raman Imaging

The same 50× objective was used for confocal Raman imaging. Areas of the protein gels, sized 200 µm × 200 µm, were scanned at 200 points per line and 200 lines per image, and spectra were collected using a laser power of 65 mW and an integration time of 1 s. These conditions were optimized based on preliminary tests to balance the signal-to-noise ratio and the time needed per image.

### 2.4. Analysis of Spectral Data and Image Processing

Raw spectra were corrected using the cosmic ray removal and background subtraction corrections described in Section 2.3.3. A manual removal of additional cosmic rays was performed using the “repair function” of the WITec Project software. In order to construct images showing the spatial distribution of the different components in the gels, the data from the Raman scans were analysed through three different methods.

#### 2.4.1. Method 1: Univariate Analysis

**Figure 1:**
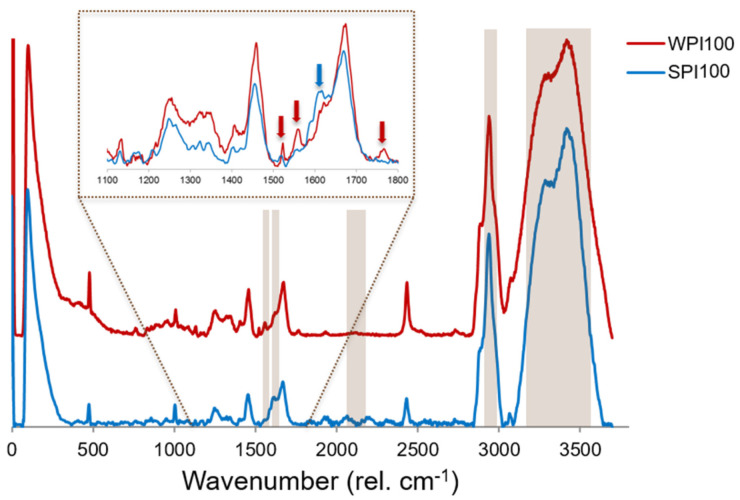
Average Raman spectra of heat-set hydrogels prepared with whey protein isolate (WPI) and soy protein isolate (SPI).

The first method was based on the integration of narrow spectral ranges (“filters”) considered of particular interest. The spectral ranges used for this analysis are given in Table 1 and highlighted in grey in Figure 1, and the criteria for their selection are discussed in Section 3.1 and Section 3.2. The intensity of each pixel in the images obtained through this method represents the area under the spectra in the selected spectral ranges at each measurement point [34].

#### 2.4.2. Method 2: Multivariate Analysis by Comparison with Reference Spectra

The second method considered the full Raman spectra, or part of it, instead of single bands to map the distribution of proteins in the gels. The reference spectra obtained, as described in Section 2.3.3, were provided as an input in the *True Component Analysis* function of the WITec Project software to map the distribution of proteins. This method assumes that the Raman spectrum obtained in each point of the sample is a linear combination of the reference spectra [34].

#### 2.4.3. Method 3: Multivariate Analysis by Automatic Detection of Components

The *True Component Analysis* function of the WITec Project software was also used to automatically detect the different components present in the protein gels. In this case, instead of inputting reference spectra to the software, it was asked to select a first component based on the most intense spectrum from the whole dataset and calculate the residual image. If the user considered that the obtained residual image was still structured (i.e., some components were not detected yet), subsequent cycles of adding components and calculating the corresponding residual images were performed, until the residual image showed just noise [34]. Figure S2 (Supplementary Materials) shows some examples of residual images to illustrate this iterative process. Once automatically detected, the spectra of the components were averaged to enhance the signal-to-noise ratio of their spectra, and manually demixed. The obtained spectra were subsequently compared with a library built from the reference spectra using the WITec True Match software.

### 2.5. Confocal Laser Scanning Microscopy (CLSM)

A total of 0.1% of fast green FCF (aq.) was added to the protein gels after sectioning with a blade, as described in Section 2.3.1. The stained samples were then observed using a Leica TCS SP5 confocal laser scanning microscope (Leica Microsystems CMS GmbH, Wetzlar, Germany). Fast green FCF was excited at 633 nm using a He/Ne laser, and the corresponding emission filter was set at 680–720 nm [20].

## 3. Results and Discussion

### 3.1. Protein Gels Preparation and CLSM Micrographs

**Figure 2:**
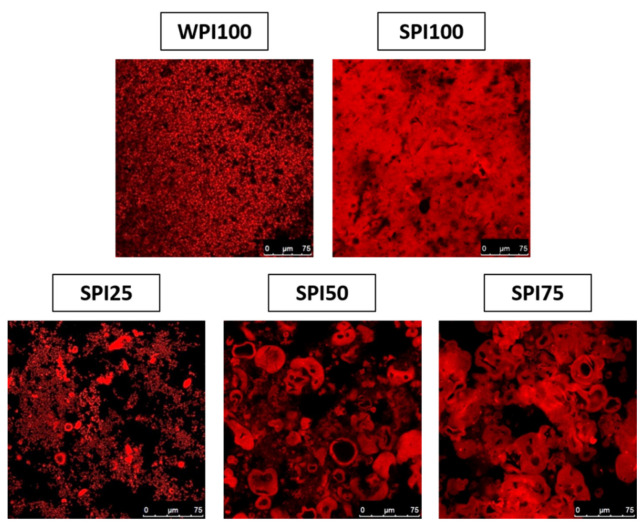
Micrographs of protein gels obtained through confocal laser scanning microscopy (CLSM).

Five different heat-set protein gels were prepared and analysed. Their characteristic microstructure is shown in Figure 2, where micrographs were obtained using a standard CLSM technique by selectively staining the proteins.

The microstructure of WPI100 was typical of WPI gels, exhibiting a porous hydrogel network formed by small protein aggregates [39]. In contrast, SPI100 showed a denser network consisting of much bigger agglomerates, consistent with the formation of microgels due to the salt-induced microphase separation of soy proteins, as described by Chen et al. [40]. These microgel structures were also observed in the mixed protein gels, especially as the SPI content increased, suggesting that the microphase separation of soy proteins also occurred in the presence of whey proteins.

The microstructure of food gels determines their mechanical properties, which, in turn, determine the texture perception of the consumer. Gels with multiple weak/fracture points tend to break apart into a larger number of small particles as compared to homogeneous or protein continuous gels, and, therefore, are perceived as more spreadable in the mouth cavity by consumers [41]. As observed in Figure 2, the phase separation of soy proteins in the mixed gels created structural heterogeneity indicative of gels that would be perceived as more spreadable.

### 3.2. Raman Spectra of Reference Protein Gels

Figure 1 shows the normalized, average spectra of heat-set protein gels prepared with the individual protein isolates, i.e., SPI100 and WPI100. The most intense bands observed in both cases were those attributed to the presence of water, centred at around 3420 and 3285 rel. cm^−1^ [35,36], given the high water content of the hydrogels. The most characteristic bands of the proteins included the ones centred at around 2939 and 1455 rel. cm^−1^, attributed to the stretching and bending vibrations of aliphatic residues (CH_3_, CH_2_, and CH groups), respectively, and the amide I and amide III bands, whose maximums were found at around 1670 and 1250 rel. cm^−1^, respectively [42,43,44]. The specific wavelengths at which the maximum of these bands was observed for each of the protein gels can be found in Table S1 of the Supplementary Material. The characteristic peak ascribed to the phenylalanine ring breathing was also observed at 1005 and 1009 rel. cm^−1^ for SPI100 and WPI100, respectively [36,42,43]. However, the intensity of other specific peaks ascribed to individual amino acid residues, generally found in the range between 500 and 1000 rel. cm^−1^ [43,44], was too low compared to the noise to unequivocally detect them due to the fact that the proteins were considerably diluted in the hydrogel systems, in which the main component was water.

Despite their similarities, some clear differences were observed between both spectra (identified by arrows in Figure 1). For instance, SPI100 gels exhibited a double peak in the area of the amide I band (blue arrow in Figure 1), while the WPI00 gels showed only a small shoulder at 1624 rel. cm^−1^, probably due to their conformational differences. Similarly, the shape of the amide III band was different for both, with the WPI100 gels having a greater relative intensity in the region above 1300 rel. cm^−1^, which is attributed to the alpha-helix secondary structures in proteins [44]. On the other hand, the WPI100 gels exhibited three distinct peaks at 1765, 1559, and 1523 rel. cm^−1^ (red arrows in Figure 1) that were not found in the SPI100 gels. Peaks at around 1550 rel. cm^−1^ were ascribed to different chemical entities in the literature, including tyrosine [45] or tryptophan [46] residues, or to the amide II band of whey proteins [42,47]. In any case, these peaks were selected as reference spectral regions for WPI in the protein gels, while the band centred at 1613 rel. cm^−1^ was chosen as the reference spectral region for SPI (Table 1 and Figure 1). The most intense bands, located at around 3420 and 2940 rel. cm^−1^, were selected as reference spectral regions for water and protein, respectively.

Another distinct feature of the SPI100 hydrogels compared to their WPI100 counterparts was that their excitation with the laser source resulted in a greater fluorescence phenomenon, which was manifested in the presence of a considerable background in their Raman spectra. Figure S1 of the Supplementary Materials shows a raw Raman spectrum for both WPI100 and SPI100 gels (i.e., before applying the background subtraction correction outlined in Section 2.3.3), where this phenomenon is more evident. Although the average spectrum, shown in Figure 1, for SPI100 displayed a flat baseline, some artefacts, due to the imperfect correction of this fluorescence phenomenon, emerged and were especially notable in the region between 1800 and 2400 rel. cm^−1^. This fluorescence phenomenon, which is usually undesirable in Raman microscopy [29], might also be exploited to help in mapping the different proteins in the mixed gels, as discussed in the following section.

### 3.3. Confocal Raman Imaging: Univariate Analysis

**Figure 3:**
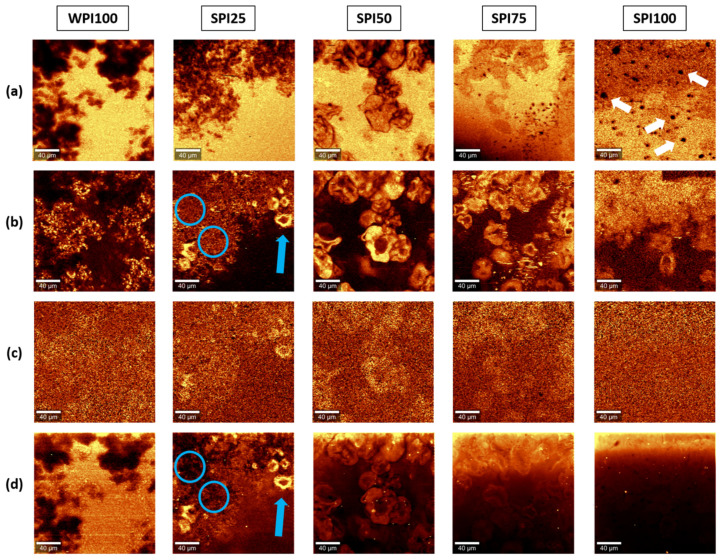
Monochromatic Raman images of protein gels obtained through univariate data analysis. Maps represent estimated distribution of water (**a**), protein (**b**), SPI (**c**), and fluorescence (**d**) obtained using the filters defined in Table 1. All scale bars correspond to 40 μm.

The spectral ranges of interest identified in the previous Section, and summarised in Table 1, were used to map the distribution of protein and water in the gels, and to attempt to identify the areas that were richer in whey or soy proteins. Taking advantage of the greater fluorescence observed in the SPI100 gels, a filter was applied in the range between 2040 and 2115 rel. cm^−1^, where no Raman scattering bands were detected, to account for differences in fluorescence in certain regions of the samples. This filter was applied to the raw datasets before applying the background correction algorithms. Figure 3 shows the monochromatic maps obtained using the selected filters.

The distribution of protein in the gels obtained through Raman imaging showed the typical structural features of heat-set WPI, SPI, and mixed proteins gels. Indeed, the images shown in Figure 3 closely resembled those obtained through CLSM (Figure 2), although using a label-free technique in this case. In general, water was located at higher concentrations, filling the areas of the gels poorer in protein, as observed for other food matrices [36]. Interestingly, SPI100 gels (and to a lesser extent, SPI75 gels) exhibited some pseudo-spherical structures devoid of water that did not correspond to regions rich in protein (see white arrows in Figure 3a). It was hypothesised that these might correspond to the presence of fat impurities, which was later confirmed in Section 3.4.2.

An attempt to distinguish between whey and soy proteins in the mixed gels was conducted by exploiting the differences between the spectra of both, highlighted in Figure 2. Although very noisy, the maps obtained using the filter for the band at 1613 rel. cm^−1^ exhibited some differences in intensity for the mixed gels. The capsule-like protein structures, previously attributed to soy protein aggregates [40], had a slightly greater intensity using this filter. However, given that both proteins exhibit some Raman scattering in that region, and that the overall protein density in those regions is higher (cf. Figure 3b), the greater intensity of these areas could not be unequivocally attributed to the presence of soy proteins over whey proteins. Nevertheless, the fact that less contrast was observed when applying that filter to pure WPI and SPI gels than in the mixed gels could be an indicative of the appropriateness of that band to preliminarily assess distribution of both types of proteins in the mixed gels. The peaks at 1765, 1559, and 1523 rel. cm^−1^ found in the WPI100 gels were also used to attempt to identify regions rich in WPI. However, the results were not conclusive (see Figure S2 of the Supplementary Materials), as each of them resulted in different distributions.

The differences in fluorescence of WPI100 and SPI100 were also used to obtain structural information of the samples. Samples SPI100, SPI75, and SPI50 were the richest in soy proteins and, therefore, were expected to exhibit the greatest fluorescence emission. However, these samples showed photobleaching, which is commonly observed for fluorescent materials and caused a considerable decrease in fluorescence during the scanning of the whole section (from top to bottom). Nevertheless, the three mixed gels (SPI25, SPI50, and SPI75) exhibited a greater fluorescence intensity in the capsule-like structures than in other protein-rich regions (see highlighted areas in Figure 3b,d, where circles show areas rich in protein but weak in fluorescence, as opposed to the arrow that identifies an area rich in protein and intense in fluorescence). This, again, supports the identification of these characteristic structures as rich in soy proteins.

### 3.4. Multivariate Analysis

Although the univariate analysis proved to be useful to map the distribution of protein and water in the hydrogels, it had some limitations in terms of distinguishing between both types of proteins due to the similarity of their spectra and the overlapping of their bands. Hence, multivariate analyses were also performed with the aim of maximizing the amount of information provided by the whole datasets.

#### 3.4.1. Comparison with Reference Materials

**Figure 4:**
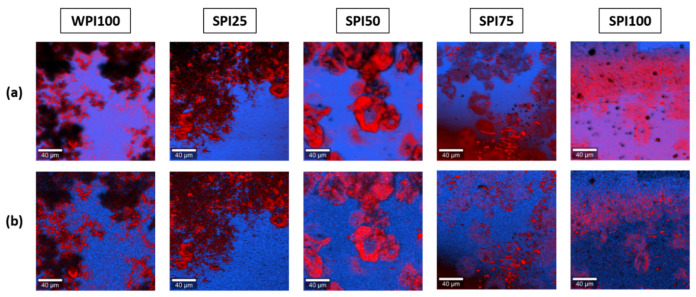
Raman images of protein gels obtained through multivariate data analysis using reference spectra. (**a**) using the whole spectra for the analysis; and (**b**) using only the region 1000–2000 cm^−1^ of the spectra for the analysis. The distributions of WPI and SPI estimated by this method are shown in red and blue, respectively. All scale bars correspond to 40 μm.

An initial analysis was performed by comparing the whole datasets obtained for each hydrogel with the average spectra of the reference gels (WPI100 and SPI100) using the True Component Analysis algorithms of the software. This methodology was previously used for the analysis of other gel systems, such as oleogels [37] and aerogels [34]. Figure 4a shows the obtained images. To avoid interference from the level of hydration of the proteins in the gels in the assignation of spectra to one of the two types of proteins, the analysis was also conduced while taking into account only the region of the spectra between 1000 and 2000 rel. cm^−1^. The results are shown in Figure 4b.

In both cases, a similar distribution of both components was obtained for the mixed gels. Therefore, including the band of water in the analysis only had a minor impact on the mapping of components, while excluding it resulted in a greater noise. Using these methods, the regions rich in protein were generally identified as rich in WPI, and the rest was identified as rich in SPI. The knowledge available from previous works contradicts these distributions, as the formation of capsule-like microgels due to salt-induced microphase separation was observed for soy proteins [40]. Moreover, the Raman images obtained for WPI100 and SPI100 hydrogels with this method showed the presence of both types of proteins in the pure gels. This demonstrated that the methodology used to obtain these images was not adequate to study these particular systems, in which both components had very similar spectra, as the presence of the two components was “forced” by the algorithms, even in the hydrogels produced with a single protein isolate.

It is also worth noting the presence of some black areas in the images obtained using this data analysis method (Figure 4). These dark regions corresponded to pixels that were not identified as rich in either SPI or WPI, which meant that the samples could not be fully described as a linear combination of the two reference average spectra. This was also reflected in their residual images, which showed a significant level of structure after extraction of both components (results not shown). Indeed, protein isolates are a combination of different proteins. Moreover, these proteins are subjected to conformational changes during formation of the heat-set gels, which can also be different in the mixed gels if these changes result in interactions between soy and whey proteins. Therefore, it was not surprising that the systems could not be mathematically explained by the linear combination of two spectra.

#### 3.4.2. Detection of Main Components without the Input of Reference Information

Using the spectra of the individual protein gels (WPI100 and SPI100) as reference to analyse the image datasets failed to provide accurate distribution of both protein isolate types. This was attributed to both the similarity of both spectra and to the possibility of conformational differences of the proteins when mixed as compared to the individual isolates. Therefore, a different approach was explored, which consisted of the detection of the different components of the samples without the input of reference information. This way, all the information was extracted from the image dataset for each specific sample, avoiding biasing the analysis with presumptions. This method, previously applied for the analysis of oleogels [37], consists of extracting the main components from the datasets one by one, using the residual image as the main criterion to decide the number of total components present in the samples. As an example, Figure S2 of the Supplementary Materials shows the residual images obtained applying this method, after extracting each of the components one by one for the image set corresponding to SPI50.

**Figure 5:**
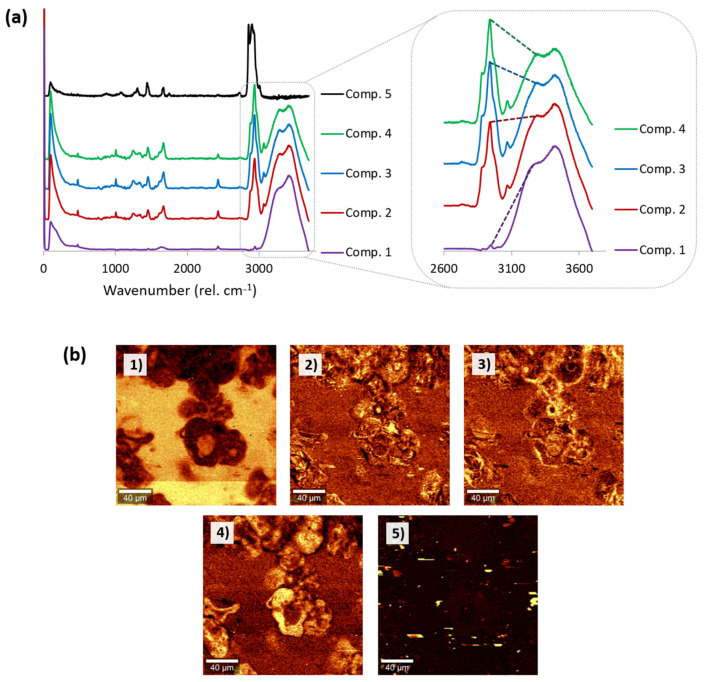
(**a**) Main components automatically detected through multivariate analysis for the mixed protein gel SPI50, and detail of 2600–3800 rel. cm^−1^ for components 1–4, highlighting differences in hydration levels. (**b**) Raman micrographs showing the distribution of each of the detected components within the gel. All scale bars correspond to 40 μm.

Using this method, between three and five different components were identified for each of the protein gels. For example, Figure 5a shows the different components identified for the intermediate protein ratio (SPI50). The spectra of most of the components identified using this method corresponded to hydrated proteins, similar to those depicted in Figure 1, and exhibiting the characteristic bands of proteins and water summarized in Table S1. The main difference between them was the level of hydration of the protein, as inferred from the varying ratio between the intensity of the band ascribed to water (ca. 3420–3283 rel. cm^−1^) and that corresponding to the stretching vibration of aliphatic residues in proteins (ca. 2940 rel. cm^−1^). Therefore, confocal Raman microscopy could be successfully used to map the distribution of protein with different levels of hydration within the gels (Figure 5b).

Interestingly, an additional component, whose spectrum was notably different from that of the hydrated proteins, was identified in all the protein gels except WPI100, that is, in all gels containing SPI. The spectrum of this component exhibited Raman bands centred at 3009, 2903, 2857, 2732, 1753, 1671, 1444, 1308, and 1081 rel. cm^−1^, all of which were identified as characteristic bands of lipids [31,37]. This confirmed the previous hypothesis that the structures devoid of water, identified in SPI-containing hydrogels in Section 3.2, indeed corresponded to fat impurities. It is worth mentioning that, even though the fat content of the protein isolates used in this study was very low (1.64% for SPI and <1% for WPI, as reported by the suppliers), the Raman signal of lipids is particularly intense compared to other food ingredients [48,49]. Therefore, Raman microscopy proved to be particularly useful to detect fat impurities in protein gels.

## 4. Conclusions

Small spectral differences were found between pure WPI and SPI gels, after background correction, due to the relatively low protein concentration in the systems and their weak Raman signal. These differences were mainly related to the amide bands of proteins, and thus attributed to conformational differences. Another distinct feature of SPI was that it exhibited considerably greater fluorescence than WPI. These differences were exploited to attempt to identify the areas richer in whey or soy proteins, using different data analysis approaches.

The univariate analysis method used, based on the integration of individual bands, provided a rapid and preliminary microstructural analysis of the gels, where the distribution of water and protein in the different gels could be successfully mapped, and was in agreement with the distribution of protein obtained by CLSM. However, no conclusive results were obtained with the univariate method of analysis in terms of distinguishing proteins from different origins. Nevertheless, the greater fluorescence intensity of the capsule-like structures found in the mixed gels compared to other regions rich in proteins suggested that these might be enriched in soy proteins.

Different multivariate analysis approaches were also used to analyse the data. The method based on comparing the datasets of each gel with the spectra of the pure SPI and WPI gels, both using the whole spectra and the 1000–2000 rel. cm^−1^ region only (to avoid interference of the level of protein hydration in their identification), was not adequate to study these systems where both components had very similar spectra, due to overfitting of the reference spectra. To avoid this bias, an alternative multivariate approach, consisting of using the datasets themselves to automatically extract the main components of the samples using the True Component Analysis algorithm, was applied. Using this method, the different levels of hydration of the proteins in the gels could be mapped, and non-proteinaceous components, identified as fat impurities, were detected and located within the protein gels.

Overall, although the distribution of whey and soy proteins in the mixed gels could not be unequivocally mapped in the mixed gels, due to the complexity of the isolates, the small spectral differences between them, and the potential conformational differences caused by interactions between proteins of both origins, the combination of univariate and multivariate data analysis methods allowed for the maximization of the amount of microstructural information obtained from Raman datasets. Confocal Raman microscopy was successfully used to map the distribution of protein with different levels of hydration and to detect lipid impurities in protein gels.

## Figures and Tables

**Table 1:** Filters used for image processing by univariate analysis.

Filter Code	Spectral Range (rel. cm^−1^)
Water	3153.6–3553.6
Protein	2907.9–2982.7
SPI	1607.1–1637.1
Fluorescence *	2040.3–2115.1

* This filter was applied to the raw dataset, before applying the background correction.

## Supplementary Materials

The following are available online at https://www.mdpi.com/article/10.3390/foods10092179/s1, Table S1: Assignments of bands from protein gels, Figure S1: Raw Raman spectra of heat-set hydrogels prepared with whey protein isolate (WPI100), soy protein isolate (SPI100), and a glass coverslip, Figure S2: Monochromatic Raman images of protein gels obtained through univariate data analysis using filters for the bands centred at 1522 rel. cm^−1^, 1561 rel. cm^−1^, and 1767 rel. cm^−1^, Figure S3: Residual images obtained for SPI50 after extracting 1, 2, 3, 4, 5, and 6 components from the dataset, using the multivariate analysis method, without the input of reference spectra.
