# Supplementary material for: Microstructural Analysis of Whey/Soy Protein Isolate Mixed Gels Using Confocal Raman Microscopy
Source: Foods. 2021 Sep 14;10(9):2179. doi: 10.3390/foods10092179 (PMC8467414; doi:10.3390/foods10092179)
Supplement: Supplementary file 1 [file foods-10-02179-s001.zip › foods-1367778-supplementary.pdf]

Supplementary Material

Microstructural analysis of whey/soy mixed protein gels using confocal Raman microscopy

Laura G. Gómez-Mascaraque, Samantha C. Pinho*

Table S1. Assignments of bands from protein gels

SPI100	WPI100	Identification
3419, 3284	3420, 3283	H_2O
3065	3074	
2937	2939	ν($-CH_3$), ν($-CH_2$), ν(-CH)
2431	2433	
1669	1672	Amide I (C=O stretch, N-H wagging)
1613	(1624)	
-	1559, 1523	(See main manuscript for potential attributions)
1455	1458	δ($-CH_3$), δ($-CH_2$), δ(-CH)
1250	1252	Amide III
1005	1009	Phe ν-ring (Phenylalanine ring breathing)

ν, stretching; δ, deformation; Phe, phenylalanine

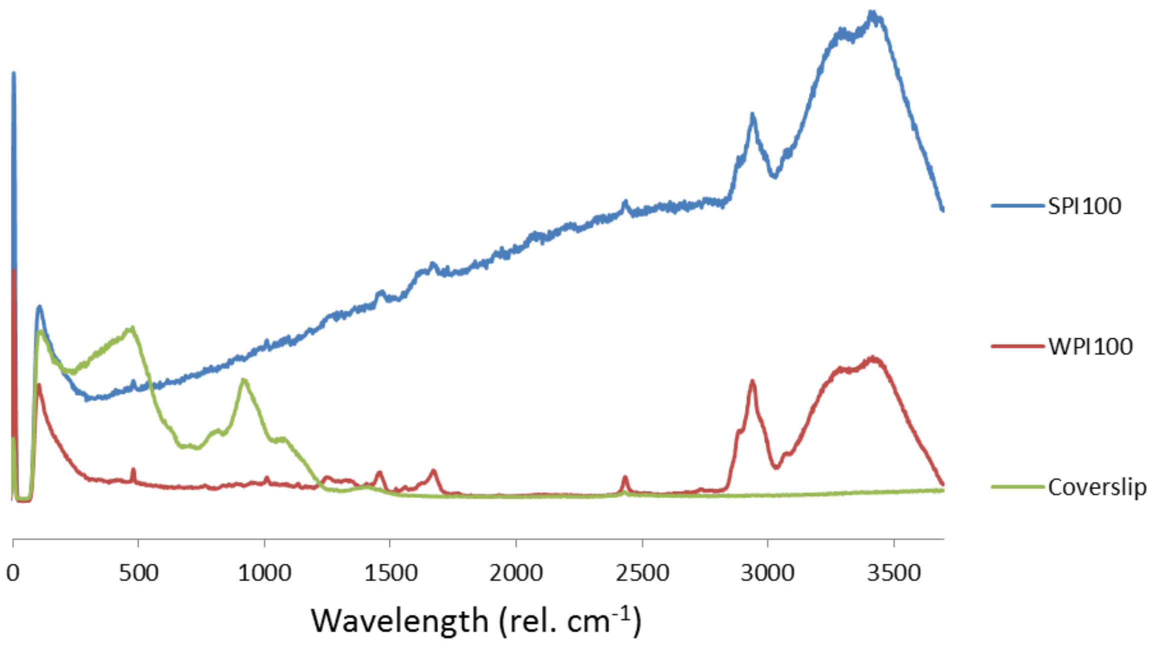

Figure S1. Raw Raman spectra of heat-set hydrogels prepared with whey protein isolate (WPI100) and soy protein isolate (SPI100), and of a glass coverslip.

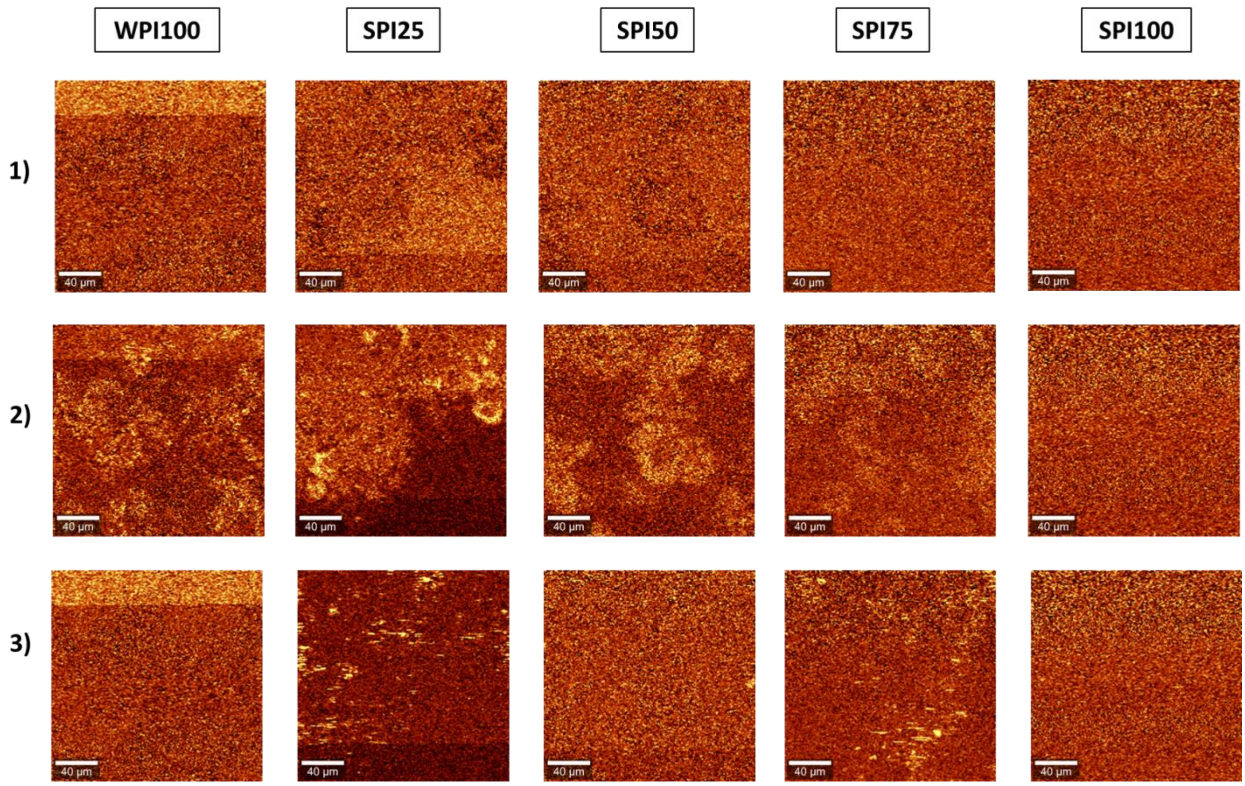

Figure S2. Monochromatic Raman images of protein gels obtained through univariate data analysis using filters for the bands centred at 1523 rel. cm^{-1} (1), 1559 rel. cm^{-1} (2) and 1765 rel. cm^{-1} (3). All scale bars correspond to 40 µm.

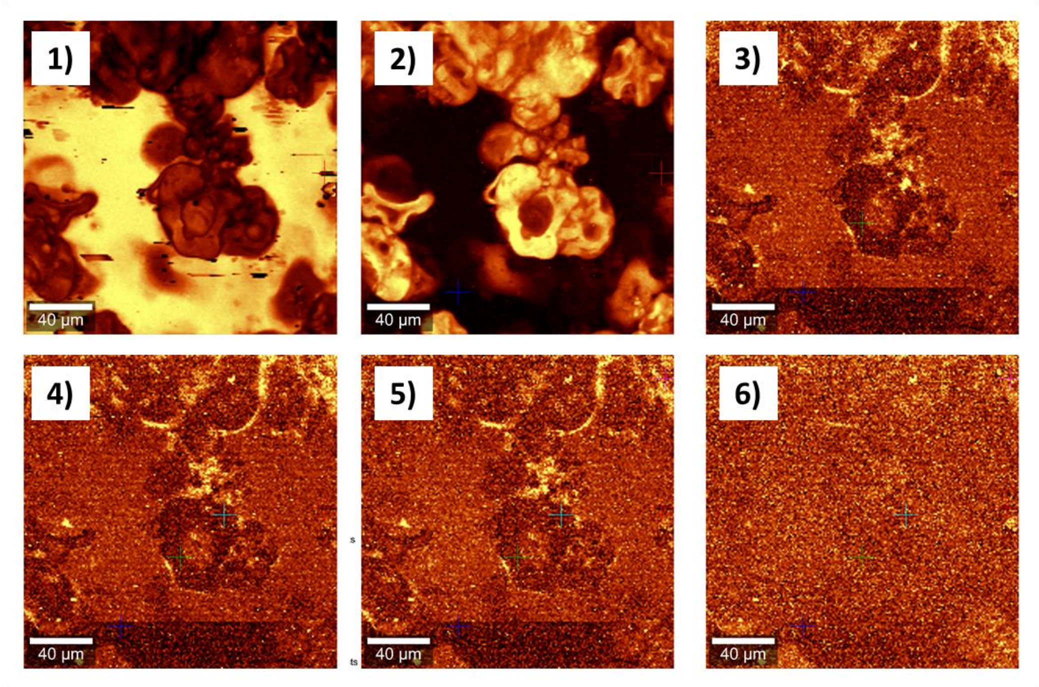

Figure S3. Residual images obtained for SPI50 after extracting 1, 2, 3, 4, 5 and 6 components from the dataset using the multivariate analysis method without the input of reference spectra
